# Vegetarianism the Radical Cure for Intemperance

**Published:** 1879-10

**Authors:** 


*Vegetarianism the Radical Cure for Intemperance.* By Harriet P. Fowler. Publishers: W. L. Holbrook & Co., New York, 1879.

The object of this work is to show that meat may lead to intemperance by its stimulating effects upon the nervous system and stomach, and by its want of carbonaceous properties.

